# A Novel Methodology for Improving Plant Pest Surveillance in Vineyards and Crops Using UAV-Based Hyperspectral and Spatial Data

**DOI:** 10.3390/s18010260

**Published:** 2018-01-17

**Authors:** Fernando Vanegas, Dmitry Bratanov, Kevin Powell, John Weiss, Felipe Gonzalez

**Affiliations:** 1 Institute for Future Environments, Robotics and Autonomous Systems, Queensland University of Technology, 2 George St, Brisbane, QLD 4000, Australia; dmitry.bratanov@qut.edu.au (D.B.); felipe.gonzalez@qut.edu.au (F.G.); 2 Agriculture Victoria Research, Victorian Department of Economic Development, Jobs, Transport and Resources, Rutherglen, VIC 3083, Australia; kpowell@sugarresearch.com.au; 3 Plant Biosecurity Cooperative Research Centre, Bruce, ACT 2817, Australia; john.weiss@ecodev.vic.gov.au; 4 Agriculture Victoria Research, Victorian Department of Economic Development, Jobs, Transport and Resources AgriBio Centre, 5 Ring Road, Bundoora, VIC 3083, Australia

**Keywords:** remote sensing, unmanned aerial vehicle, phylloxera, multispectral, hyperspectral, RGB, digital elevation model, digital vigour assessment

## Abstract

Recent advances in remote sensed imagery and geospatial image processing using unmanned aerial vehicles (UAVs) have enabled the rapid and ongoing development of monitoring tools for crop management and the detection/surveillance of insect pests. This paper describes a (UAV) remote sensing-based methodology to increase the efficiency of existing surveillance practices (human inspectors and insect traps) for detecting pest infestations (e.g., grape phylloxera in vineyards). The methodology uses a UAV integrated with advanced digital hyperspectral, multispectral, and RGB sensors. We implemented the methodology for the development of a predictive model for phylloxera detection. In this method, we explore the combination of airborne RGB, multispectral, and hyperspectral imagery with ground-based data at two separate time periods and under different levels of phylloxera infestation. We describe the technology used—the sensors, the UAV, and the flight operations—the processing workflow of the datasets from each imagery type, and the methods for combining multiple airborne with ground-based datasets. Finally, we present relevant results of correlation between the different processed datasets. The objective of this research is to develop a novel methodology for collecting, processing, analysing and integrating multispectral, hyperspectral, ground and spatial data to remote sense different variables in different applications, such as, in this case, plant pest surveillance. The development of such methodology would provide researchers, agronomists, and UAV practitioners reliable data collection protocols and methods to achieve faster processing techniques and integrate multiple sources of data in diverse remote sensing applications.

## 1. Introduction

Recent advances in remote sensed imagery and geospatial image processing using unmanned aerial vehicles (UAVs) have enabled the creation of rapid and ongoing monitoring tools for crop management [1,2,3,4] and the detection/surveillance of insect pests. However, there are still challenges in remote sensing applications, such as detecting early incursions of cryptic pest species such as grape phylloxera (*Daktulosphaira vitifoliae* Fitch) in vineyards. Grape phylloxera is currently present in most grape-growing countries, but relatively localised in wine districts in southeastern Australia. Grape phylloxera is a very small insect that primarily lives underground, feeds on the roots of the grapevines, and consequently damages the root system. This creates impairing water and nutrient uptake to the plant, which causes stress that is expressed above-ground by impairment and changes in photosynthesis, changes in pigment ratios, reduced canopy, slow stunted growth, and reduced yield [5]. The symptoms of infestation appear usually after two to three years, although in some instances this can be longer [6].

The current surveillance practice for growers is to visually inspect their grapevines during December to April for signs of grapevine damage attributable to a phylloxera infestation [7]. A more intensive method of monitoring utilises phylloxera emergence traps at a density of one trap every fifth panel of every third grapevine row (standard trapping method) [8]. Traps are deployed in the spring and summer months, when immature (first instar) insects move from the grapevine roots onto the soil surface [7]. The traps are usually placed at the base of suspected infested grapevines and inspected after two to four weeks. In addition, on suspected infested grapevines, the roots can be inspected for yellow galls, swellings on the older roots, and the yellow insects [9]. Although these practices have been extensively used as standard methods, they are time consuming, season dependent, labour intensive and require taxonomic expertise [10].

Ground-based spectral observations using hyperspectral imagery have been conducted to characterise the spectral response of phylloxera-infested stressed grapevines [11]. However, this study focused only on leaf-level observations. In addition, several aerial studies have been conducted for phylloxera detection and monitoring [12,13,14]. A canopy-level characterisation of phylloxera has been studied [15], but further research needs to be conducted to develop reliable predictive detection methods. Typical indices for analysis of stress vegetation include the normalised difference vegetation index (NDVI) and colour infrared composite (CIR) [7], but they are not able to distinguish the stress caused by phylloxera infestation from the stress caused by other sources, and may only be useful in monitoring temporal changes in phylloxera distribution over known infested vineyards [16]. Aerial thermal imagery can be used to identify grapevines showing indications of yellowing or decline that require closer inspection [17].

A spatial and temporal analysis methodology has been applied for grapevine vigour analysis to assess the health of a vineyard [18]; however, hyperspectral imagery was not considered. Micro UAV have been used for generating georeferenced orthophotos for precision agriculture purposes, with the aim of producing vegetation indices in the visible (VIS) and near-infrared (NIR) spectrum [19], yet there is a need for higher spectral resolution imagery to produce different vegetation indices. UAV-based cloud point and hyperspectral imagery systems have been developed and used for a biomass estimation of wheat and barley crops [20,21] but this new technology still needs to be tested in broader applications. Low altitude aerial imagery has been trialed to detect diseases in avocado trees using different vegetation indices from RGB images [22]. UAV and aerial RGB imagery have also been applied to the detection of infestation symptoms on olive trees (canopy discolouration) and palm trees mapping [23], but only visible symptoms are considered, and further research needs to be to done to include vegetation indices and spectral signatures analysis. 

This paper presents the methodology for developing a predictive detection method for pests using processed airborne RGB, multispectral, and hyperspectral data combined with ground-collected data. Among the methods used for processing the data are the photogrammetry of RGB imagery, the development of an airborne RGB-based digital elevation model in order to assess the canopy vigour, the georeferencing of different datasets, the processing of multispectral and hyperspectral imagery to generate vegetation indices, and the extraction of mean spectral signatures of grapevines for different levels of pest infestation at the canopy level. 

Section 3 describes the UAV and sensors used for airborne imagery collection. Section 4 describes the field experiments carried out in December 2016 and February 2017.

Finally, we show results for the correlation of the most relevant vegetation indices to expert vigour assessment and a digital vigour model, and provide conclusions on these preliminary findings and insights on the ongoing and further work required to generate a predictive model for pest infestation in crops (in this case grapevine phylloxera). A second paper will focus on verifying and validating a predictive model of a case study of a serious pest in vineyards. We envision the title to be Improving Plant Health Surveillance in Vineyards and Row Crops: a Predictive Model for the Early Detection, Extent, and Impact of Grape Phylloxera*.*

## 2. Methods

### 2.1. Predictive Detection Model Workflow

**Figure 1:**
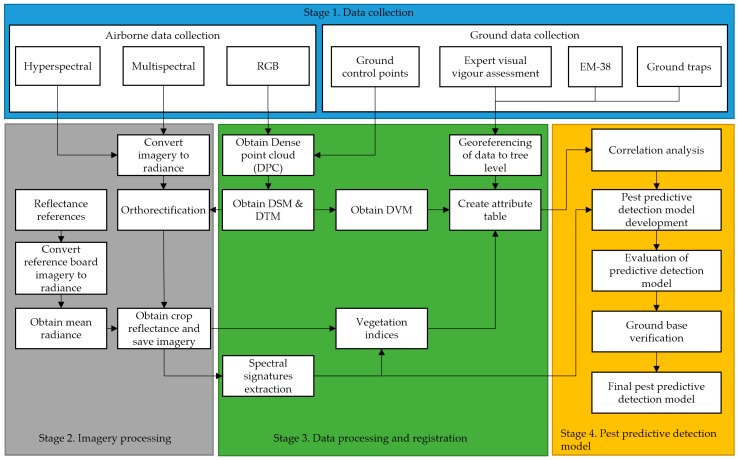
Predictive detection model workflow.

Our approach for generating a predictive model for pest and diseases in vineyards or other row crops e.g., avocado trees, macadamia trees, etc., employs multiple stages (Figure 1). The first stage of the process is data collection. This includes the collection of airborne RGB, multispectral and hyperspectral imagery, and ground data in the form of ground control points (GCP), reflectance references, expert visual vigour assessment, EM-38 soil conductivity, and ground traps counts.

In the second stage, multispectral and hyperspectral images are processed to obtain radiance. All of the imagery was created with data input from ground control points, and using structure from motion algorithms is orthorectified. This process generates orthomosaics of the entire crop. Multi and hyperspectral orthomosaics are processed to obtain reflectance data cubes using mean white references obtained from boards and imagery taken in pre and post-flight operations. 

The next stage uses these reflectance images to extract spectral signatures of the crop at the canopy level, with different levels of infestation, for the calculation of numerous vegetation indices that are selected based on the symptoms of infestation (e.g., reduced chlorophyll content, leafs yellowing). Digital surface models (DSM) and digital terrain models are obtained from the RGB orthomosaics in order to produce a digital vigour model (DVM).

The next step in this stage is to combine all of the multiple sources of data into a single information system. In order to do this, there is a segmentation process to obtain data from individual plants, with the aim of creating a table that contains different attributes for a single plant within the crop. The result of the previous step is an attribute table containing the extracted values of several vegetation indices (see Table 1), expert vigour assessment classes, and values estimated from a DVM. The table also consists of traps data that is only used for the verification of suspected infestation, and EM38 soil conductivity data that was collected on the field, which could highlight differences in soil moisture and thus possible regions where phylloxera could establish.

The following is the list of the attributes used in the georeferenced table:

1. Tree number
2. Latitude
3. Longitude
4. Block
5. Row
6. Panel
7. Tree variety
8. Expert visual vigour assessment
9. Digital vigour model
10. Multispectral derived indices and bands
11. Hyperspectral derives indices and bands
12. EM38 data

Once the table is populated with georeferenced data, the next stage in the process is the development of a pest predictive detection model. The first step in this stage is to carry out a correlation analysis between the expert vigour assessment as ground truth, and the vegetation indices calculated using the multispectral and hyperspectral data, the EM38 data, and the DVM. 

The results obtained in the correlation analysis are the foundation for the development of a preliminary phylloxera detection model, which is followed by an evaluation and a ground-based verification to obtain a final phylloxera detection model. This final stage is out of the scope of this paper, and is part of ongoing research work.

### 2.2. Orthorectification, Hyperspectral Imagery Processing, and GIS Tools

Data orthorectification of colour and multispectral data was conducted using Agisoft Photoscan (Agisoft LLC., St. Petersburg, Russia). The software allowed us to perform the photogrammetric processing of digital images and to generate three-dimensional (3D) spatial data including 3D models, orthomosaics, and digital elevation models (DEM).

The photogrammetry orthorectification process starts with the alignment of photos, where the software refines the camera positions of each photo and builds a sparse and dense point cloud model of objects in the multiple collected airborne images. Then, key points are projected onto the selected reference system. Finally, ground control points (GCP) with highly accurately observed geolocations can then improve the sparse point cloud.

We processed the hyperspectral data with Headwall SpectralViewer, MATLAB and Scyllarus^®^ DATA61 Matlab open source toolbox. Scyven software from DATA61 was used for analysing and cropping the white reference (this process is explained in Section 2.4) for transforming radiance data cubes into reflectance [24]. Matlab was used to produce the different indices, run a first approach for pixel classification based on these indices, generate plots of the phylloxera spectral response, and process the hyperspectral data cubes to obtain radiance.

The ArcMap 10.5 (ESRI, Redlands, CA, USA) software was used to incorporate the different sources of data into several layers, in order to analyse and visualise the data. It was also used to extract the spectral signatures from the grapevines that are next to the phylloxera traps, and to generate the attribute table generating vegetation indices for every plant.

Google Earth enabled us to visualise overlaid imagery obtained from indices and phylloxera traps, as well as to distinguish the boundaries of the different grapevine varieties in the crops.

### 2.3. Georeferencing

The georeferencing using GCP was conducted and reported using a free online AUSPOS service managed by Geoscience Australia, and took less than 10 min for a GCP. We included GCPs into the georeferencing of multispectral and colour data after the generation of a sparse points cloud, followed by camera locations optimisation and dense point cloud generation routines. 

### 2.4. Ground Control Points (GCP) and White Reference Boards

We refined the georeferencing process of the collected imagery using custom-made GCPs with high contrast ground markers and applied precise point positioning (PPP) localisation methods to geolocate them. We used a triple frequency Novatel DL-V3 GPS receiver with a Novatel 703 antenna and Geoscience Australia online service in order to get the localisation of GCPs to a 3 cm accuracy level; whereas standard single frequency GPS chip in Canon/MicaSense sensors typically provided positioning accuracy within 5–10 m. 

**Figure 2:**
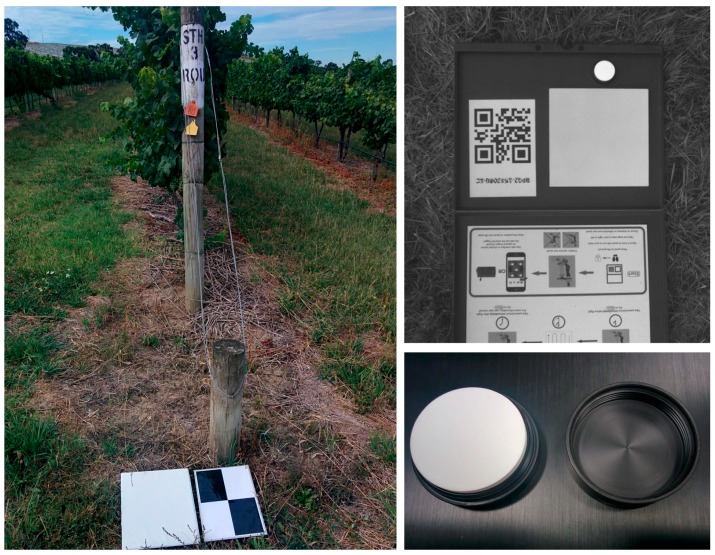
Ground control point (GCP) target and white reference board (**left**), MicaSense reflectance reference board (**top right**), and Spectralon white reference (**bottom right**).

Additionally, we deployed a set of ground reflectance references in order to calculate reflectance in the multispectral and hyperspectral imagery. A MicaSense reflectance reference board (grey reference) and a 100 percent white reference Spectralon target were used before and after each flight as primary calibration tools for the cameras. We also deployed a set of nine white reference boards to the sites to be able to adjust reflectance in case of changing lighting conditions during the flight. Figure 2 shows the GCP, white reference boards located aside, and the grey and white reflectance references.

**Figure 3:**
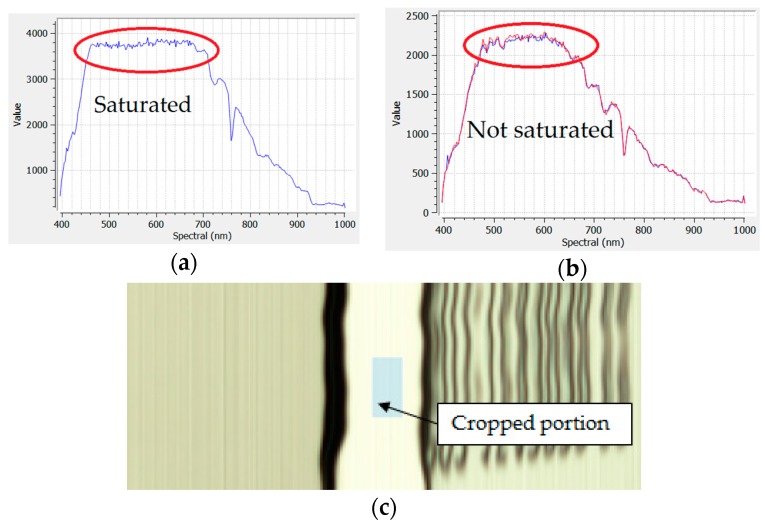
Hyperspectral white reference processing. (**a**) Saturated values around the highlighted area; (**b**) Maximum values are not saturated and this white target portion can be used for obtaining reflectance; (**c**) Cropped portion of the white reference board image with higher radiance values.

Images of the white reference boards were taken using the hyperspectral camera before and after the flights on the field ground. The hyperspectral images of the white reference board were first verified to avoid using images that have saturation in some of their bands (Figure 3a,b), and the portion of the image with the higher radiance values is cropped (Figure 3c). The aerial images were then divided by the mean radiance value of the bands extracted from the cropped white reference image to obtain the reflectance of the surveyed field.

### 2.5. Vigour Assessment

Vigour assessment is conducted both visually by an expert and using orthorectified images to generate a digital vigour model. Estimating and analysing crop and grapevines vigour from orthorectified imagery when the crop or vineyard is on sloping terrain where elevation changes significantly over a small area is a challenging task. Even within one row of grapevines, it is possible to see elevation changes that are much more intense than the height of the grapevines. This means that using the terrain’s flat surface as a reference to estimate the vigour and height of individual grapevines is not accurate, and a better method is required. 

Our approach was to generate dense point clouds (DPC), and classify these dense points to at least two classes: grapevines and terrain. We ran an optimisation of the DPC (*camera = 10 px, marker accuracy = 0.001 m, marker placement = 0.1 px).* We additionally controlled the reprojection error of generated DPC at level below 2 px. As a segmentation criteria in classification, we used a set of geometrically defined restrains, which we optimised experimentally. We set *max distance = 0.6 m*, i.e., limited a distance between the point in question and terrain model, and set *max angle = 70 deg*, which limited the maximum slope of the ground within the scene. Then, we built two types of meshes and DEM. One set will form a digital surface model (DSM). The second set uses the rest of the DPC augmented with inferred surfaces constructed by closing the gaps under the grapevines. The latter forms a bare-earth or digital terrain model (DTM). We then subtracted the elevations of the DTM from the DSM to get a digital vigour model (DVM) in order to analyse and better represent differences of vigour. 

In this way, we generated unbiased models of grapevines’ height or vigour. Then, we localised grapevine trunks based on ground data, and performed a zonal analysis of each grapevine within a 0.2 m radius from the trunk. 

### 2.6. Hyperspectral Processing

We implemented the workflow described in Figure 1 (Stage 2) to pre and post-process the airborne collected hyperspectral data. First, the hyperspectral data cubes are transformed from digital numbers into radiance using the Headwall hyperspec software. Second, we orthorectify each of the scans using the GPS time-stamps from the flight. We then generated orthomosaics by stitching together multiple scans. Finally, the reflectance for the orthomosaic data cubes was determined by dividing the radiance data cubes by the mean radiance of the white target boards that are located in the field during the survey flights.

### 2.7. Mean Spectral Signatures for Different Grapevine Types

**Figure 4:**
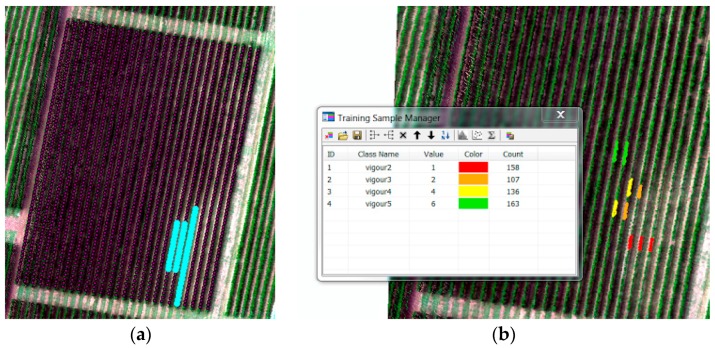
Selection of individual plants or grapevines for spectral signature extraction based on vigour assessment into four classes. (**a**) Selected grapevines from an attribute table where vigour is 2/5 (**b**) Four different polygon areas for each class based on grapevine location and vigour assessment.

We extracted the mean spectral signature for plants with different values of the Modified Cab Absorption in Reflectance Index (MCARI) index. In ArcMap 10.5, we selected polygons containing the pixels of a single crop or grapevine. Figure 4 shows an example of the process where different regions are selected for the signature extraction. Grapevines within the red, orange, and yellow areas show signs of phylloxera infestation, whereas grapevines within the green region are healthy.

### 2.8. Vegetation Indices

We calculated vegetation indices from multispectral and hyperspectral imagery. The indices used were selected in order to evaluate symptoms of infestation such as the premature yellowing of leaves and a reduction in chlorophyll content. A list of the indices used, as well as their equations, is found in Table 1, where index H stands for hyperspectral data and M stands for multispectral data, respectively.

We also created six new indices based on the analysis of the spectral reflectance of infested and uninfested crop grapevines. The two distinct spectral responses were subtracted to highlight the main differences and explore relevant bands with high differences and bands with equal reflectance. In this way, we detected seven relevant spectral bands, from which we created indices PI1 to PI6, as described in Appendix A, Equations (A1)–(A6). 

## 3. UAV and Sensors

The methodology described in Section 2 is platform or sensor agnostic if the UAV platform or sensor has similar characteristics. For the purpose of this work, we demonstrate the methods using the following:

### 3.1. UAV

The UAV used is an S800 EVO Hexacopter (DJI Ltd., Shenzhen, China) weighing 6.0 kg and capable of taking an additional payload up to 2.0 kg. The frame is fitted with a retractable undercarriage, providing a sensor field of view clear of obstacles. The UAV uses a 16,000 mAh LiPo six-cell battery, which provides a maximum hover time of approximately 20 min with no sensor payload. A WooKong-M flight controller forms the navigation and control system of the UAV, and comes with a stabilisation controller, a GPS unit with an inbuilt compass, and an inertial measurement unit (IMU). The flight controller has multiple autopilot modes to enable both remote control by operator and autonomous go home/landing with an enhanced fail-safe operation following a payload-specific predefined flight path, position and altitude hold.

### 3.2. High Resolution RGB Camera

A Canon 5DsR camera (Canon Inc., Tokyo, Japan) was integrated into the S800 UAV to capture ultra HD colour (RGB) GPS-stamped images. The camera features the latest full-frame 50.6-megapixel CMOS sensor with Dual DiGIC 6 processors and a 28-mm Canon lens. Specific flight patterns were flown to enable processing the images using photogrammetry software (e.g., Agisoft Photoscan) in order to generate comprehensive geospatial orthomosaics, 3D models, and DEM. The sensor brings to our study an ability to generate remarkable spatially detailed models of the crop in order to look after the spatial component of the infestation symptoms.

### 3.3. Multispectral Camera

We used a multispectral MicaSense RedEdge camera (MicaSense Inc., Simi Valley, CA, USA) to capture five discrete spectral bands: 475 nm (blue), 560 nm (green), 668 nm (red), 717 nm (red edge), and 840 nm (NIR). The sensor combines 1.2-megapixel CMOS spatial capability and two additional NIR and red edge spectral bands. The images produced by this camera were orthorectified and stitched together to produce rasters that were used to generate vegetation indices e.g., NDVI. Additionally, this camera supported the generation of DEM with resolutions of 3.26 cm/px for 60 m flights and 6.74 cm/px for 100 m flights.

### 3.4. Hyperspectral Sensor

**Figure 5:**
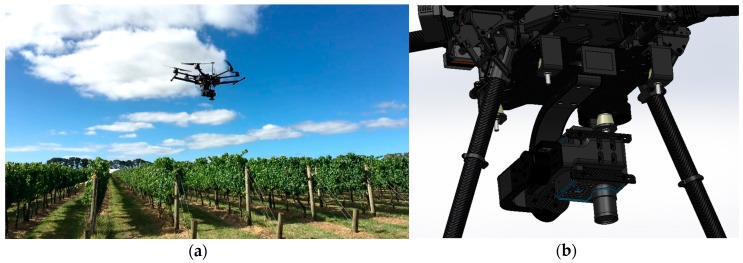
(**a**) Headwall Nano hyperspectral sensor on-board a S800 unmanned aerial vehicle (UAV); (**b**) Custom-made two-axis gimbal hosting the hyperspectral camera (SolidWorks 3D model).

Hyperspectral imagery was acquired using a Headwall Nano-Hyperspec (Headwall Photonics Inc., Bolton, MA, USA). The hyperspectral sensor recorded data cubes of 274 spectral bands in the visible and near-infrared (VNIR) range (400–1000 nm) with a ~2.2 nm spectral interval and a 5-nm spectral resolution (full width at half maximum (FWHM) with 20 μm slit). This camera is equipped with a calibrated f/1.8 4.8 mm Schneider lens, which results in a 50.7 deg field of view over 640 pixels. The collected hyperspectral data cubes are synchronised with GPS/inertial navigation system (INS) positioning and orientation information in order to perform data cubes orthorectification and multiple data cubes mapping. The hyperspectral sensor was integrated into a S800 UAV using a custom designed gimbal done by the Queensland University of Technology (QUT) Research Engineering Facility (REF). This gimbal has two axes, which ensures the seamless operation of the push-broom hyperspectral scanner in windy conditions. The QUT REF gimbal design features carbon reinforcement over a 3D printed structure, advanced dampening, brushless motors, and a BaseCam SimpleBGS 32 bit gimbal controller, with the total weight below 1 kg. Mounting the push-broom scanner on the gimbal enhances the camera performance in high turbulence environments by ensuring consistent and minimal required overlaps between consecutive data cubes over the large study area. This leads to an increased overall flight efficiency in open environments. Figure 5a shows the S800 UAV with the hyperspectral sensor on the gimbal during one of the missions. The gimbal computer assisted design (CAD) model is presented in Figure 5b.

### 3.5. Expert Visual Vigour Assessment and EM-38 Data

In addition to the UAV airborne data and GCP, the methods use ground truth data for confirming the presence of grape phylloxera. The ground truth data included ground traps for insect presence and abundance, and, when necessary, digging to confirm the presence of the insect on the roots, expert visual vigour assessment, and EM38 soil conductivity. Table 2 shows an example of the vigour assessment classification for phylloxera-infested grapevines used in the visual assessment performed by the expert on the field.

## 4. Field Experiments

Two major aerial surveys were carried out at two locations during December 2016 and February 2017. Different sensors were deployed to collect comprehensive datasets. Our objective was to evaluate diverse state of the art remote sensing capabilities, and its combinations, in order to evaluate the methodology for the early detection of phylloxera infestations. This section describes the different aerial platforms and sensors used, and how the data was collected.

**Figure 6:**
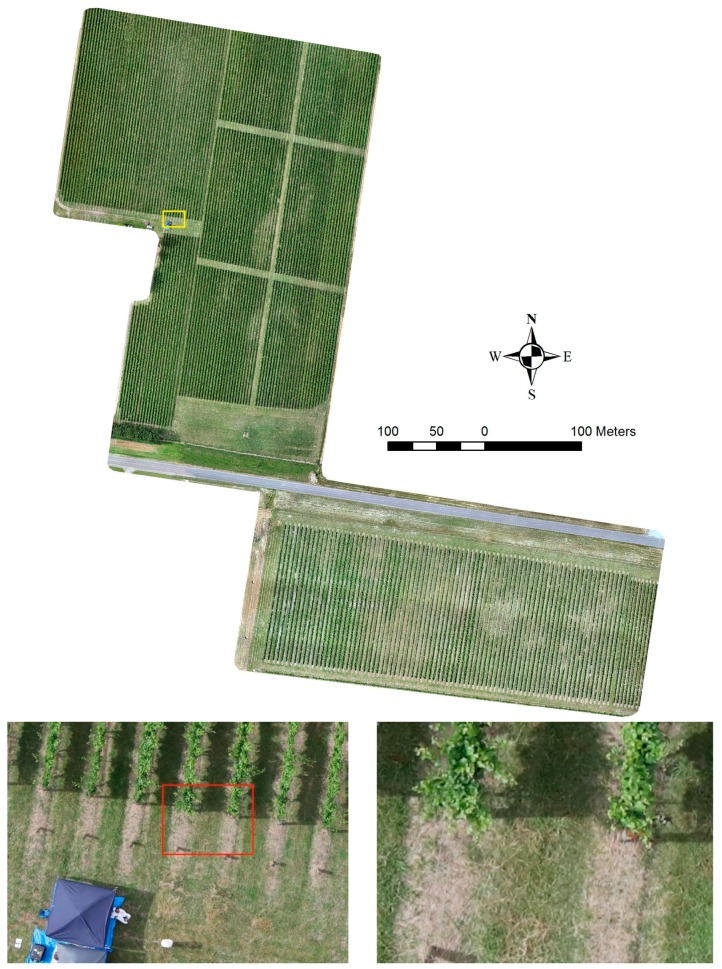
Generated orthomosaic of studied sites; two vineyards in the Yarra valley, Victoria, Australia. Bottom images show enlarged regions of the vineyard with clearly visible plants.

Two phylloxera-infested vineyards with multiple grapevine varieties in Yarra Valley, Victoria, Australia were surveyed (see Figure 6). These two sites were selected because they have been continuously monitored by Victorian Department of Economic Development, Jobs, Transport and Resources (DEDTR) personnel over several grapevine-growing seasons. Site one included 104 rows of ungrafted *V. vinifera* cultivars Chardonnay, Pinot Noir, Shiraz, and Merlot over an area of 8.5 ha containing 160 to 266 grapevines per row. Site two included 80 rows of ungrafted *V. vinifera* cultivars Cabernet Sauvignon, Pinot Noir, Merlot, and Roussanne, with 59 to 63 grapevines per row over 3.16 ha. In this paper, we present our analysis on a single block within one vineyard containing the Chardonnay variety, and some remarkable results from different blocks for method verification purposes.

**Figure 7:**
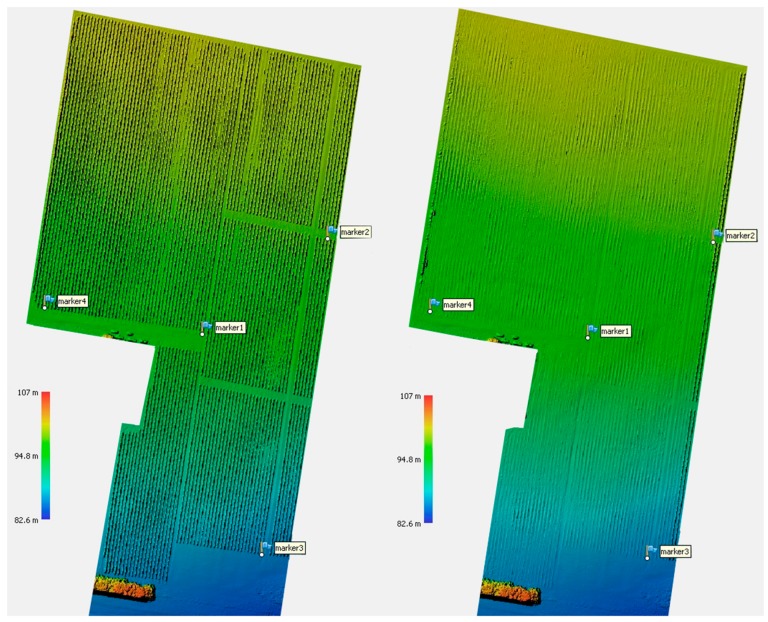
Result of the digital surface model (DSM) and digital terrain model (DTM) of Site 1. Markers are the ground control points (GCP).

We placed eight GCP over the sites for an hour, which lead to a horizontal positional uncertainty below 0.014 m, and a vertical uncertainty below 0.046 m (95% confidence levels, GDA94) over the array of points. Figure 7 denotes the location of four GCP as marker flags distributed over site 1.

In the studied vineyard, the top, middle, and bottom supportive wires were positioned at approximately 1.75 m, 1.55 m, and 1.2 m (average values for the two studied vineyards) above the ground, respectively. They were used as an approximate guidance to generate the canopy vigour classes (see Table 2).

The imagery was acquired on two consecutive days during the grapevine phenological cycle: post-flowering (14–15 December 2016) and veraison (14–15 February 2017). This approach is common [7,11], and enables temporal studies on the identification of phylloxera symptoms and population development, as well as validation.

## 5. Results and Discussion

### 5.1. Visual Vigour Assessment Results

**Figure 8:**
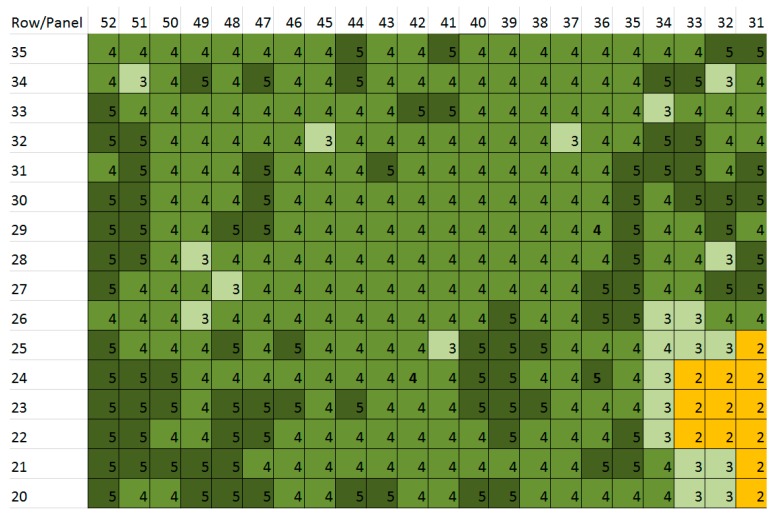
Results of traditional expert-based assessment of grapevines vigour per panel (groups of four to six grapevines, fragment transposed to cardinal directions).

The result of the expert’s visual vigour assessment (Figure 8) is a matrix of vigour health, with the cell size equal to the size of the panel and distance between rows. Therefore, the expert-based dataset is spatially sparse, and not aligned geographically. Ground-based vigour assessment is time consuming, and only evaluated every 3–5 m. The expert observations indicated the start of another weak spot (low vigour) at row 48–50, panels 26–28.

### 5.2. Digital Vigour Model (DVM)

The results of the generated DVM allowed us to classify the grapevines according to its vigour using classes from 2 to 5 from Table 2. In this study, the two surveyed vineyards did not contain plants with low vigour (class 1). 

**Figure 9:**
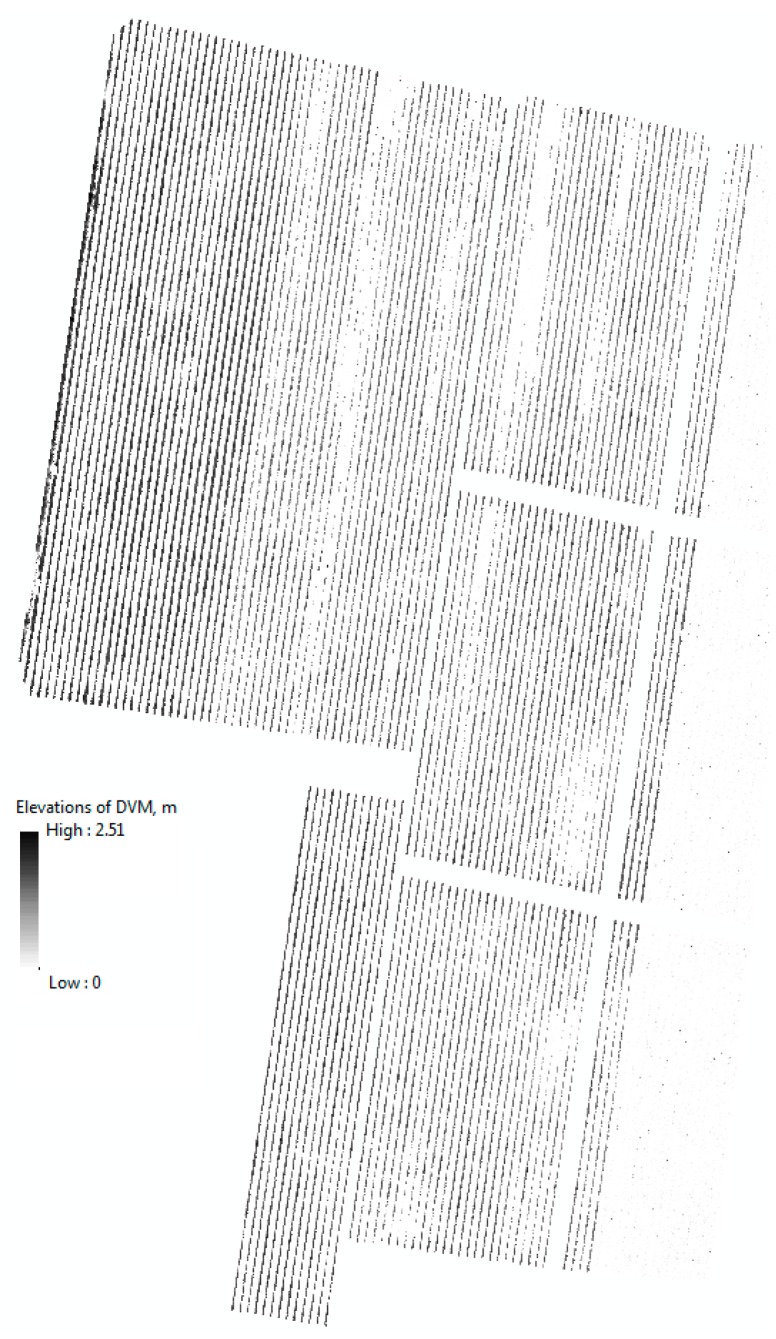
Result of the unbiased DVM for Site 1.

**Figure 10:**
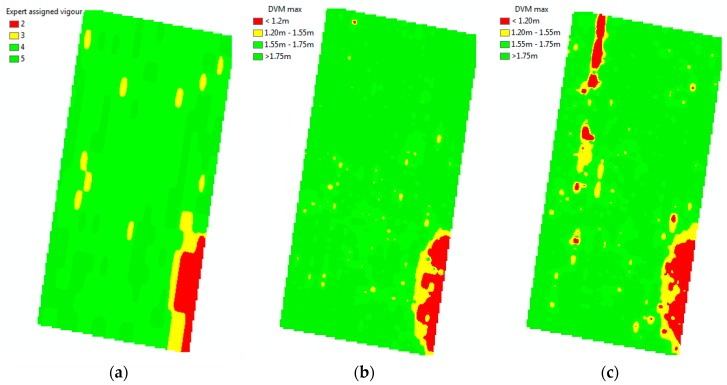
Expert assigned classes of grapevines vigour per panel (**a**) and the results of a remotely-sensed vigour assessment of individual grapevines for December 2016 (**b**) and February 2017 (**c**) for a block with the Chardonnay variety.

**Figure 11:**
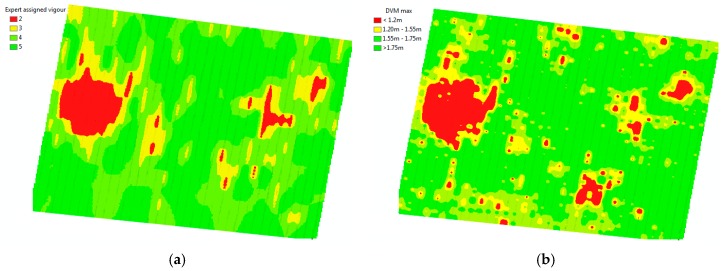
Expert-assigned classes of grapevines vigour per panel (**a**) and the results of a remotely-sensed vigour assessment of individual grapevines for February 2017 (**b**), site 2 with the *V. vinifera* Roussanne variety.

The result of the DSM and DTM show a range of elevations on the surface and terrain of approximately 24 m (see legend of DSM and DTM for site 1 on Figure 7). The result of the technique described in the methods section for the DVM shows that we reduced that range of elevations within the studied area to 2.5 m (Figure 9, Figure 10 and Figure 11), which corresponds well to tree heights.

Figure 9, Figure 10 and Figure 11 show the georeferenced results of an expert’s vigour assessment Figure 10a and Figure 11a) and derived DVM (Figure 10b,c and Figure 11b) (inverse distance weighted interpolation) for the two sites. The season average Pearson coefficients of a grapevine’s expert-assigned vigour versus DVM remains at 0.396, but mostly correlated at the time of actual vigour assessment (0.414) for the area at site 1 and (0.52) site 2. We note a specific feature in the infestation extension, which tends to happen along the rows from one infested grapevine to another, whereas the transition of the disease between rows is less likely. 

The zonal statistics method combines simplicity and numerous statistical parameters for the buffered zones. The Pearson correlation coefficient *r* = 0.52 between an expert-based assessment (Figure 8) and max values of the DVM for individual grapevines as a result of remote sensing for the described area (Figure 11b) for site 2 demonstrates an adequate correlation and validity of the used method.

Geolocalised results (Figure 10b,c) give a number of advantages over standard expert-based assessments: much more spatially accurate localisation (<0.02 m), individual tree assessment rather than panel approximation, and the ability to detect subtle trends that are unnoticeable due to inaccurate references used for visual assessment, such as the height of the poles. One of the most important advantages is the shorter time required to assess a large area. However, the inability to detect early symptoms of the disease and occasional irregular behaviour of the affected grapevines (early infested grapevines sometimes demonstrate abnormal intense vigour for a short time) are the known limitations of the DVM method. Nevertheless, the DVM method could provide insight to the expert to select candidate zones to revisit for closer and more detail inspections.

### 5.3. Hyperspectral Analysis

Different vegetation indices were computed on the vineyard imagery in order to highlight the symptoms of the phylloxera infestation, which include a reduction in plant vigour and the reduction of chlorophyll content. 

**Figure 12:**
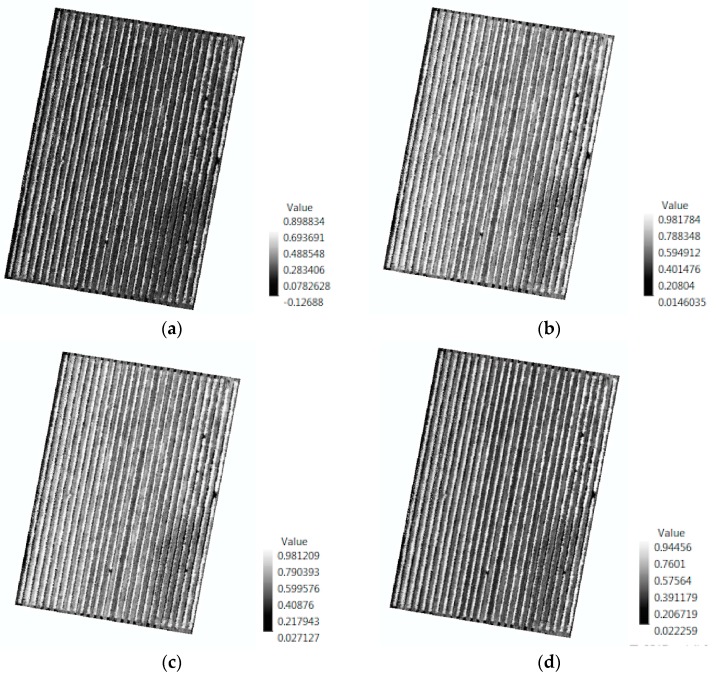
Vegetation indices for block 3 with the highest correlation to vigour assessment. (**a**) PI2; (**b**) PI5; (**c**) NDVI; and (**d**) OSAVI_H_. All of the indices are based on hyperspectral imagery collected in February 2017.

Figure 12 shows examples of four vegetation indices calculated from hyperspectral data for block 3, namely PI2, PI5, NDVI, and OSAVI_H_.

**Figure 13:**
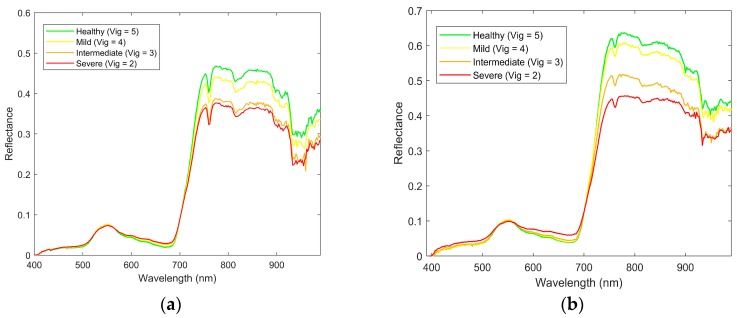
Mean spectral signature for different levels of vigour of the grapevine for the *V. vinifera* Chardonnay variety measured in (**a**) December 2016; and (**b**) February 2017 for wavelengths from 400 to 1000 nm.

**Figure 14:**
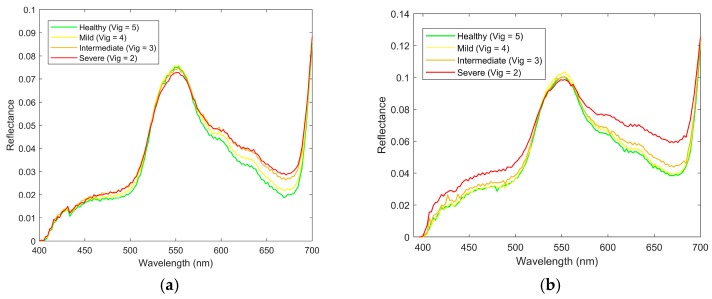
Mean spectral signature for different levels of vigour of the grapevine for the *V. vinifera* Chardonnay variety measured in (**a**) December 2016 and (**b**) February 2017 for wavelengths from 400 nm to 700 nm.

The result of the mean spectral signature extraction is shown in Figure 13a,b. Notice the different signatures of the grapevines that are affected by phylloxera. The infested grapevines show higher reflectance in the visible region, and lower reflectance in the NIR region. Furthermore, infested vines have higher levels of reflectance at the chlorophyll well around 670 nm, with the healthy grapevines absorbing more light around this wavelength (Figure 14a,b). Spectral signatures also show higher differences between infested and uninfested grapevines for the February 2017 imagery. 

**Figure A1:**
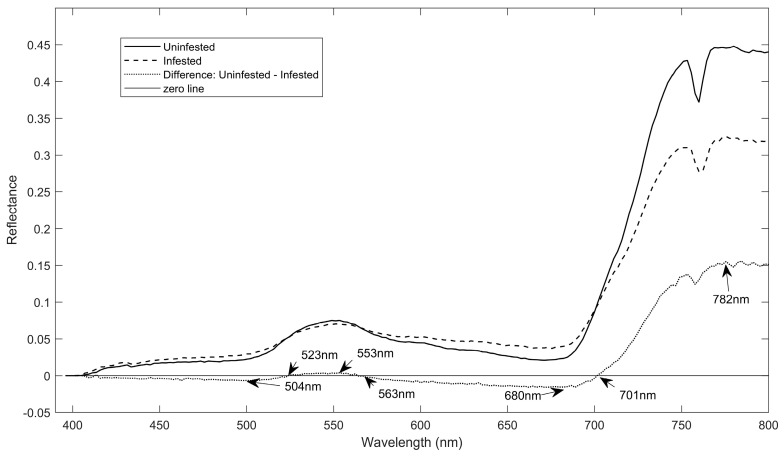
Mean spectral signature for different levels of vigour of the grapevine for the Chardonnay variety measured in December 2016 for wavelengths from 400 nm to 700 nm.

Figure A1 in Appendix A shows the difference in spectral responses for infested and uninfested grapevines for the *V. vinifera* Chardonnay variety. Spectral bands of interest are marked with arrows. These bands correspond to local points where the difference in the reflectances is either high or zero.

### 5.4. Correlation Analysis of Different Variables Using Attribute Tables

**Figure 15:**
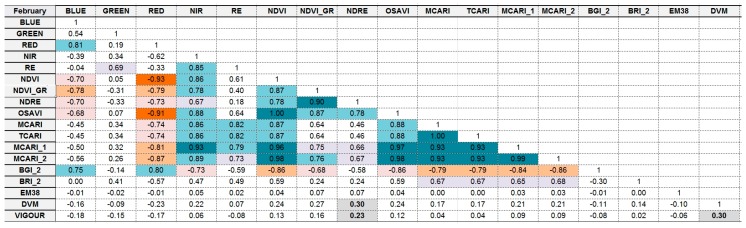
Pearson’s correlation coefficient matrix showing the strength of the relationship between the February 2017 multispectral vegetation indices and expert and digital vigour assessment. Orange colours indicate negative correlation, blue colours indicate positive correlation, and intensity of colour indicates relative strength.

**Figure 16:**
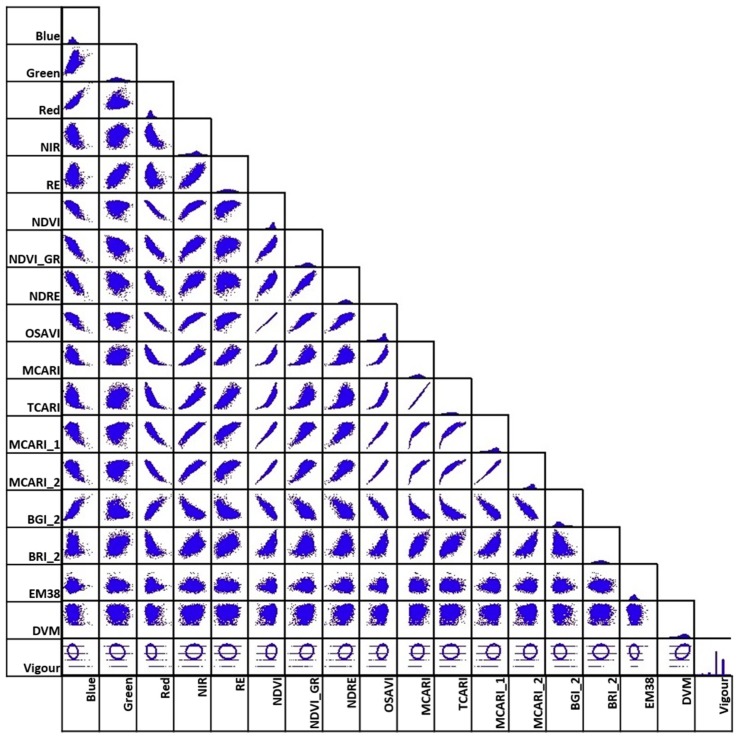
Scatter plot of correlation for data presented in Figure 15, generated from multispectral imagery in February 2017.

Figure 15 and Figure 16 show the results of calculating Pearson’s correlation to indices and bands extracted from multispectral imagery. In particular, we calculated correlation among the following data: blue, green, red, NIR, and red edge bands, and NDVI_M_, NDVI_GreenM_, Normalised Difference Red Edge (NDRE_M_), OSAVI_M_, MCARI_M_, Transformed Chlorophyll Absorption in Reflectance Index (TCARI_M)_, MCARI_1M_, MCARI_2M_, Blue/Green Index (BGI_2M_), Blue/Red Index (BRI_2M_), EM-38, DVM, and expert visual vigour assessment (Vigour). 

We found the following cross-correlations between analysed multispectral-based indices:

1. Vigour in both December 2016 and February 2017 has the highest positive correlation with DVM, and to NDRE_M_, but only to NDVI_M_, NDVI_GreenM_, and OSAVI_M_ in December. However, these are relatively minor relationships, with the linear relationship between 0.23 and 0.3.
2. EM38 has no relationship to any of the vegetation indices, Vigour, or DVM.
3. Certain indices are extremely positively correlated for December and February (indicated by dark blue boxes in Figure 15). These are: BLUE, GREEN, RED, and Red Edge (RE) multispectral bands; NIR with RE bands, OSAVI_M_, MCARI_M_, TCARI_M_, MCARI_1M_, and MCARI_2M_; NDVI_M_ with GREEN, NDRE, OSAVI_M_ and MCARI_M1_; NDVI_GreenM_ with NDRE and OSAVI_M_; and TCARI_M_ with MCARI_M_, MCARI_1M_, and MCARI_2M_.

**Figure 17:**
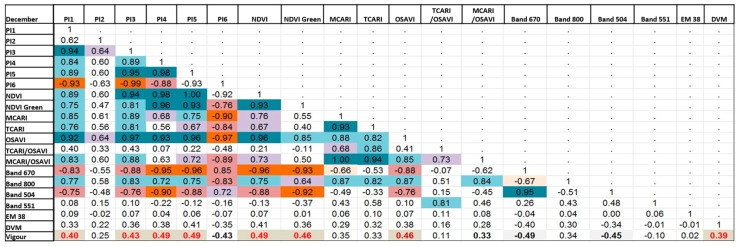
Pearson’s correlation coefficient matrix showing correlation between the hyperspectral vegetation indices and expert vigour assessment for data collected in December 2016. Orange colour indicates negative correlation; blue colour indicate positive correlation with the intensity of colour indicating relative strength and highlighted red font are the indices that correlate positively with the digital vigour model (DVM) and expert vigour assessment.

**Figure 18:**
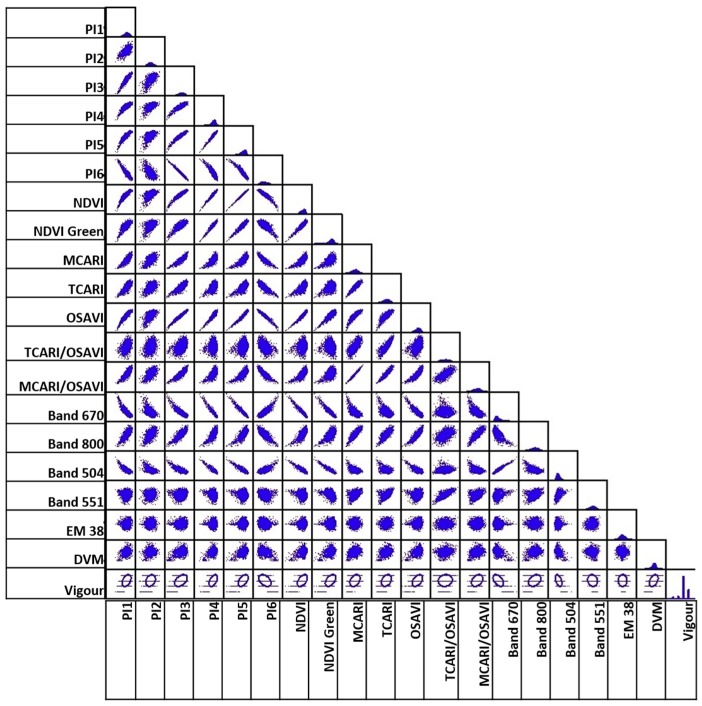
Scatter plot of correlation for data presented in Figure 17, generated from hyperspectral imagery in December 2016.

Most of the newly created hyperspectral vegetation indices showed higher correlations to vigour and to DVM (Figure 17 and Figure 18) compared with the multispectral indices. This might be due to the difference in the spectral resolution of the cameras. Using the hyperspectral camera with a higher spectral resolution, we were able to distinguish specific bands or points of interest such as the ones shown in Figure A1, and thus generate indices that are not possible to create with the multispectral camera.

1. Vigour showed the highest positive relationship (*r* > 0.4) with the vegetation indices PI1, PI3, PI4, PI5, NDVI, NDVI_GreenH_, and OSAVI, as well as with DVM (*r* = 0.4).
2. Similar to the multispectral data, certain vegetation indices were positively correlated for both December and February; PI1, PI3, PI4, PI5, NDVI, NDVI_GreenH_, MCARI_H_, TCARI_H_, and OSAVI_H_ with each other; MCARI/OSAVI with BAND800; and BAND670 with BAND504.
3. The EM38 data showed no relationship with any of the vegetation indices, vigour, or DVM.

Overall, the correlation values of the vegetation indices compared with the vigour assessment are moderate, but some important relationships were found. This was expected, because those are different symptoms that a stressed plant manifests in response to a phylloxera infestation, but in different ways depending on the different stages of infestation and environmental factors. Moreover, some indices might better highlight specific changes in the leave reflectance than others, depending on the specific bands used. Furthermore, the values of the indices are calculated averaging the reflectance for all of the pixels of the tree canopy coverage. This technique allows us to sample every plant within a block in the vineyard.

## 6. Conclusions and Further Research

In this work, we presented the methodology for processing airborne-collected imagery using RGB, multispectral, and hyperspectral cameras for assessing the condition of vineyards with the aim of generating a predictive model for the detection of pests in crops and vineyards. The methods are demonstrated for detection of phylloxera in vineyards. In particular, we presented the implementation of a digital vigour model, the evaluation of several vegetation indices, and the creation and evaluation of new indices based on hyperspectral signatures to highlight symptoms of grapevine phylloxera infestation.

We presented the results of comparing a digital vigour model of the vineyard to an expert visual assessment. We found that the two assessments correlate positively indicating that the developed method is a correct approach for generating vigour assessments in vineyards.

We generated vegetation indices to highlight possible symptoms of phylloxera infestation, such as changes in the reflectance/absorption of light indicating a reduction in the chlorophyll content in the grapevines. This could be done remotely using both the multispectral and hyperspectral collected and treated imagery.

Furthermore, we identified mean spectral signatures for different levels of infestation for the Chardonnay variety at two different times of the year which helped us find regions of interest in the spectrum in order to generate new vegetation indices to highlight grape phylloxera infestation. 

The development and use of a specific phylloxera or existing vegetation index is important to vineyard managers for two reasons. Initially, such an index could be used on imagery collected from an infested vineyard to determine the extent (area) and severity of the plant pest and its impact on grape production. This would also aid in vineyard management decisions, such as where to implement hygiene protocols. The second reason is that this index can improve the potential for early detection of the pest. At present, the existing surveillance methods (visual vine and root inspection and emergence traps) are ineffectual at detecting early infection of the vines by grape phylloxera. 

We have shown that hyperspectral imagery has the potential to detect grape phylloxera before it is apparent to visual inspection. The next stage is to determine if these hyperspectral indices have the potential to be adapted to the multispectral data without a loss in correlative power. Future work will focus on deriving predictive models (utilising these indices) and testing their accuracy in predicting the presence/abundance of the plant pest. A paper describing the development and validation of the predictive model (Stage 4 of Figure 1) is in preparation as a complement of this paper.

The methods, workflow, results and analysis presented in this research will contribute to the generation of valuable information for plant pest surveillance. The presented methodology could also be extrapolated to other areas of research in remote sensing, such as minerals exploration, biodiversity, and ecological assessment.

## Figures and Tables

**Table 1:** Vegetation indices used in the analysis of the phylloxera infestation in vineyards.

Vegetation Index	Equation	Reference
Normalised Difference Vegetation Index (NDVI)	NDVI_H_ = (R_800_ − R_670_/(R_800_ + R_670_) NDVI_M_ = (R_840_ − R_668_/(R_840_ + R_668_)	[25]
Normalised Difference Vegetation Index (NDVI_Green_) (Green band)	NDVI_Green H_ = (R_800_ − R_551_/(R_800_ + R_551_) NDVI_Green M_ = (R_840_ − R_560_/(R_840_ + R_560_)	
Normalised Difference Red Edge (NDRE)	NRDE_M_ = (R_840_ − R_717_)/(R_840_ + R_717_)	[26]
Modified C_ab_ Absorption in Reflectance Index (MCARI)	MCARI_H_ = [(R_700_ − R_670_) − 0.2(R_700_ − R_551_)] (R_700_/R_670_) MCARI_M_ = [(R_717_ − R_668_) − 0.2(R_717_ − R_560_)] (R_717_/R_668_)	[27]
Modified Chlorophyll Absorption in Reflectance Index (MCARI_1_)	MCARI_1 M_ =1.2 [2.5 (R840 − R668) − 1.3(R840 − R560)]	[28]
Modified Chlorophyll Absorption in Reflectance Index (MCARI_2_)	${\mathrm{MCARI}}_{2M}=\frac{1.5\left[2.5\left(R_{840}-R_{668}\right)-1.3\left(R_{840}-R_{560}\right)\right]}{\sqrt{{\left(2R_{840}+1\right)}^{2}-\left(6R_{840}-5\sqrt{R_{668}}\right)-0.5}}$	[28]
Transformed CARI (TACRI)	TCARI_H_ = 3[(R_700_ − R_670_) − 0.2(R_700_ − R_551_)(R_700_/R_670_)] TCARI_M_ = 3[(R_717_ − R_668_) − 0.2(R_717_ − R_560_)(R_717_/R_668_)]	[29]
Optimised Soil-Adjusted Vegetation Index (OSAVI)	OSAVI_H_ = (1 + 0.16)(R_800_ − R_670_)/(R_800_ + R_670_ + 0.16) OSAVI_M_ = (1 + 0.16)(R_840_ − R_668_)/(R_840_ + R_668_ + 0.16)	[30]
Blue/Green and Blue/Red Pigment indices	BGI_2 M_ = R_475_/R_560_ BRI_2 M_ = R_475_/R_668_	[31]
Phylloxera index 1 (PI1)	PI1 = (R_522_ − R_504_)/(R_522_ + R_504_)	(This study)
Phylloxera index 2 (PI2)	PI2 = (R_551_ − R_562_)/(R_551_ + R_562_)	
Phylloxera index 3 (PI3)	PI3 = (R_700_ − R_680_)/(R_700_ + R_680_)	
Phylloxera index 4 (PI4)	PI4 = (R_782_ − R_700_)/(R_782_ + R_700_)	
Phylloxera index 5 (PI5)	PI5 = (R_782_ − R_671_)/(R_782_ + R_671_)	
Phylloxera index 6 (PI6)	PI4 = (R_680_ − R_563_)/(R_680_ + R_563_)	

**Table 2:** Phylloxera vigour classes from an expert visual assessment.

Class	Vigour	Criteria	Phylloxera Presence Conjecture
5	High	Plant or grapevines to or above a given height e.g., top supportive wire	Healthy (probably no infestation e.g., phylloxera)
4	Medium-high	Plant just below a given height e.g., top supportive wire	Mild symptoms (probably no infestation or early stages of impact e.g., phylloxera)
3	Medium	Plant height below middle wire and above bottom wire	Intermediate impact (probably low levels of infestation e.g., phylloxera)
2	Low	Short plants e.g., grapevines. Plants below bottom wire	Severe symptoms of infestation (e.g., phylloxera, surrounding (3–4) plants also likely to be infested)
1	No vigour	Dead plant	Extreme symptoms of infestation (e.g., phylloxera has been affecting the plant for years)

## Appendix A

New Phylloxera Indices Creation

A new phylloxera index was created by first selecting some interest points are chosen for creating indices looking at the difference between the spectral signatures of the infested and the uninfested grapevines (Figure A1). We then selected the wavelengths that have local minimum and maximum and zero values for reflectance in the difference spectral signature.

Seven bands of interest were found which wavelengths are: *R*_504_, *R*_523_, *R*_553_, *R*_563_, *R*_680_, *R*_701_ and *R*_782_.

From these seven spectral bands, the following indices were created to highlight the main differences between the uninfested and infested spectral signatures.

The phylloxera infestation indices concentrates on highlighting the differences between the pairs of spectral bands *R*_504_ and *R*_523_ (Equation (A1)), *R*_551_ and *R*_562_ (Equation (A2)), *R*_700_ and *R*_680_ (Equation (A3)), *R*_782_ and *R*_700_ (Equation (A4)), *R*_782_ and *R*_671_ (Equation (A5)) and bands *R*_680_ and *R*_563_ in Equation (A6):

(A1) $$PI1=\;\frac{R_{522}-R_{504}}{R_{522}+R_{504}},$$

(A2) $$PI2=\;\frac{R_{551}-R_{562}}{R_{551}+R_{562}},$$

(A3) $$PI3=\;\frac{R_{700}-R_{680}}{R_{700}+R_{680}},$$

(A4) $$PI4=\;\frac{R_{782}-R_{700}}{R_{782}+R_{700}},$$

(A5) $$PI5=\;\frac{R_{782}-R_{671}}{R_{782}+R_{671}},$$

(A6) $$PI6=\;\frac{R_{680}-R_{563}}{R_{680}+R_{563}}.$$
